# Percutaneous mechanical thrombectomy using the Rotarex^®^S device for the treatment of acute lower limb artery embolism: A retrospective single-center, single-arm study

**DOI:** 10.3389/fsurg.2022.1017045

**Published:** 2023-01-06

**Authors:** Wenrui Li, Yunchao Xing, Hai Feng, Xueming Chen, Zhiwen Zhang

**Affiliations:** Department of Vascular Surgery, Beijing Friendship Hospital, Capital Medical University, Beijing, China

**Keywords:** acute lime embolism, acute lower limb ischemia, percutaneous mechanical thrombectomy (PMT), catheter-directed thrombolysis (CDT), endovascular

## Abstract

**Objective:**

Acute limb embolism (ALE) is a challenging, highly morbid, and frequently fatal vascular emergency. Percutaneous mechanical thrombectomy (PMT) devices are an alternative treatment to restore perfusion by removing emboli in the limb arterial system. We evaluated the outcomes of treatment of ALE patients using PMT devices in our center.

**Methods:**

A retrospective review of ALE patients treated with Rotarex S (Straub Medical) at a single institution from 2018 to 2022 was performed. The primary outcome was technical success, defined as complete recanalization of the occluded segment with satisfactory outflow and good capillary filling of the distal parts of the foot without any major or obstructing residual emboli or thrombi either in the treated segment or in the outflow tract without the need for additional catheter-directed thrombolysis (CDT) or conversion to open surgery. Embolized segments treated, treatment outcomes, and perioperative complications were reviewed.

**Results:**

A total of 17 ALE patients (29% men, 71% women; mean age, 73 years) underwent PMT procedures. The femoral arteries and popliteal arteries are the most commonly treated vessels, with both present in 59% of the patients. The technical success rate was 100%, but the majority of cases (82%) had concurrent percutaneous transluminal angioplasty or stent grafting, and two patients were treated with urokinase during the operation. There was one thrombotic recurrence that required amputation. There were no 30-day deaths. Complications included extravasation after PMT (two), intraoperative embolization of the outflow tract (one), access site hematoma (one), target artery thrombosis (one), and acute kidney injury (one). There were no severe bleeding complications.

**Conclusions:**

The Rotarex S device has a satisfactory success rate, although complementary use of various adjunctive techniques is frequently required. It seems to be a moderately effective tool for treating ALE to avoid CDT or open surgery. The device appears safe, with low risks of amputation and mortality rates, but special attention should be given to the potential for extravasation and distal embolism.

## Introduction

Acute limb ischemia (ALI) occurs when there is sudden reduced arterial perfusion to a limb, mostly owing to thrombosis or emboli. When left untreated, it can threaten the viability of the limb, giving rise to life-threatening complications related to systemic metabolic conditions (1, 2). As mentioned above, acute limb embolism (ALE) is one of the common causes of ALI, which mostly comes from a cardiac origin in 88%–90% of cases and usually occurs in patients with cardiac arrhythmias, such as paroxysmal atrial fibrillation or recent myocardial infarction (3, 4). In addition, abdominal aortic aneurysms, ulcerated atherosclerotic plaques with thrombi, and, rarely, venous sources with paradoxical embolisms are other potential sources of macroembolism (5).

Fast restoration of appropriate flow to preclude limb loss and other complications is required in these situations (6). Compared with patients treated electively for peripheral arterial disease, ALE patients are not medically optimized and have more advanced disease, which contributes to significantly higher postoperative complications, mortality, and limb loss (7). The therapeutic approach depends on the severity and duration of symptoms associated with limb ischemia at presentation (8). The European society for vascular surgery (ESVS) published the guidelines for ALI patients. Conventional open surgeries, such as embolectomy/thrombectomy or bypass and/or catheter-directed thrombolysis (CDT), have been first-line procedures, and immediate revascularization is required for patients with Rutherford grade IIb acute limb ischemia (6, 9). New endovascular therapies have been developed in the past decade that aim at percutaneous thrombus and emboli extraction. Techniques including percutaneous mechanical thrombectomy (PMT) or pharmacomechanical thrombectomy and ultrasound-accelerated thrombolysis (USAT) are performed as alternative choices (1, 10).

Among other thrombectomy devices on the market, Rotarex S (Straub Medical) was used in our institution for several years in different acute ischemia situations, including superior mesenteric artery embolism or renal artery thrombosis, with safe and satisfactory results (11, 12). In this study, we evaluated the outcomes of the treatment of ALE patients using the Rotarex S device in our center.

## Methods

This was a retrospective study of ALE patients treated with Rotarex S at our center from March 2018 to February 2022. We identified 17 consecutive patients who met the inclusion criteria, and no patients were excluded from the study. Their medical records were subsequently reviewed, and arterial duplex ultrasound or arteriography was recorded to confirm the diagnosis of ALE and identify the occluded arterial segments. The severity of ALI was determined based on the Rutherford classification. Data are expressed as proportions for dichotomous variables and as the mean ± SD or median and interquartile range (IQR) (25–75th percentiles) for continuous variables. All analyses were performed using SPSS 24.0 software (SPSS Inc., Chicago, IL, United States).

To assess vessel patency, an innovative classification called TIPI (thromboaspiration in peripheral ischemia) was used (13). The primary outcome was a technical success. It was defined as TIPI 2–3 flow (near complete or complete revascularization of the occluded artery with any distal outflow) without additional CDT or conversion to open surgery. The degree of residual stenosis was obtained by reviewing angiography images and reports. Secondary outcomes included treatment complications, continuous limb ischemia, amputation, and mortality 30 days after surgery. All of the additional treatment modalities were also recorded.

## Procedure description

**Figure 1:**
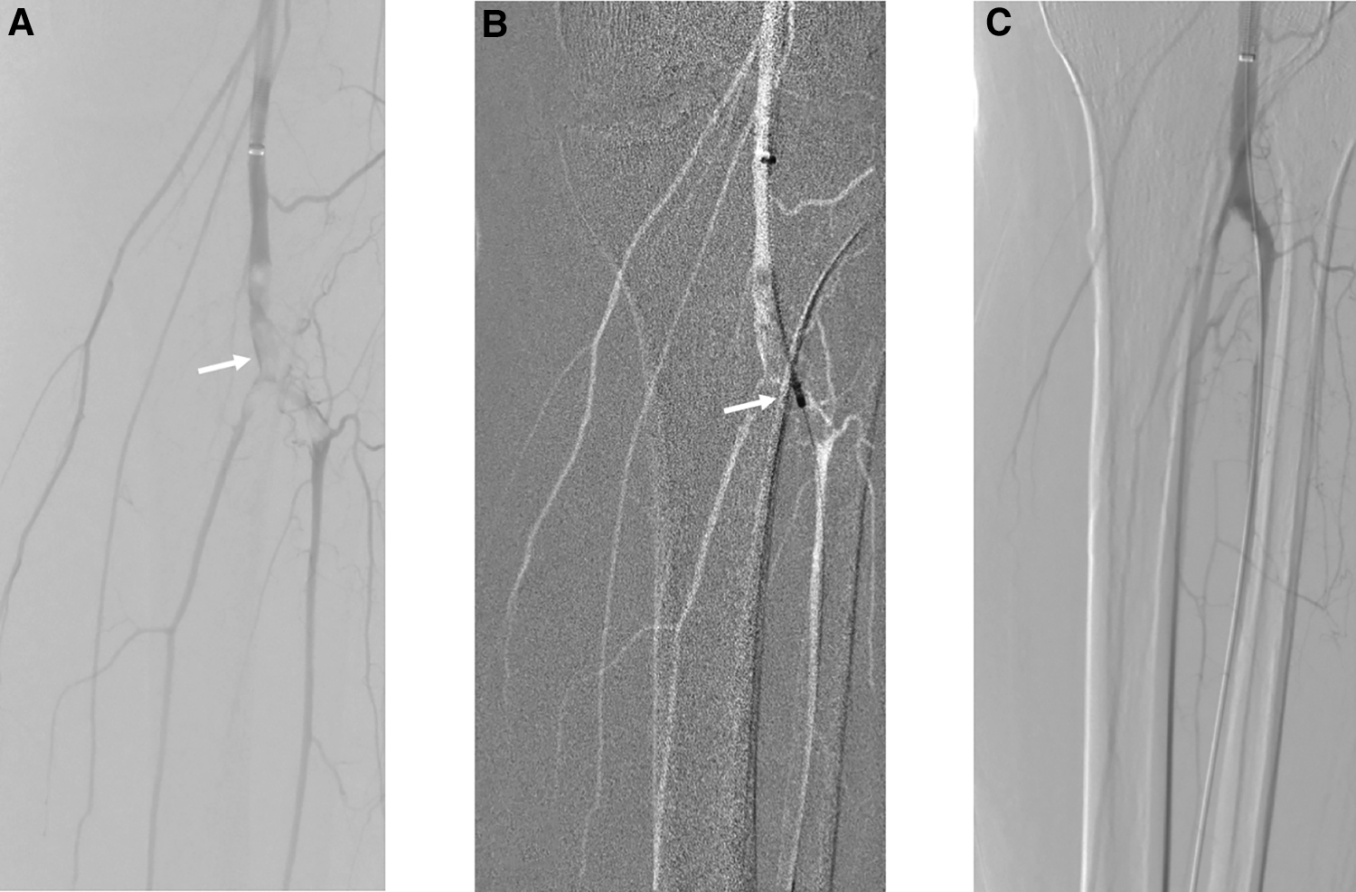
Acute embolism of popliteal and tibial arteries treated by mechanical thrombectomy with Rotarex S, PTA, and intraoperative urokinase. (**A**) White arrow points to the embolism of popliteal and tibial arteries. (**B**) PMT with Rotarex S under the roadmap. (**C**) Final result of recanalization after PTA and intraoperative urokinase.

All ALE patients were evaluated by a proceduralist (vascular surgeon or interventional radiologist) to decide the mode of therapy. In general, elderly patients or those with a decreased degree of ischemia were thought to be more favorable to treat with this device. All operations were performed in a hybrid operating room under local anesthesia combined with moderate sedation. Systemic anticoagulation was administered as in any other angioplasty procedure. Percutaneous access was chosen on the basis of clinical examination, orientation duplex ultrasound, and CT angiography, and the size of the access site depended on which Rotarex S aspiration thrombectomy catheter (Straub Medical, Wangs, Switzerland) was planned to be used (6F/8F). After the embolized segment was traversed with a V-18 ControlWire guidewire (Boston Scientific, Natick, MA, United States), it was then replaced with a special 0.018-inch, 260-cm-long wire matching with the Rotarex S system. Over this wire, mechanical thrombectomy was performed with a flushed Rotarex S system, and small, careful forward and backward passages were slowly performed once or twice. Completion angiography was performed to assess the treatment results (Figure 1). Additional percutaneous transluminal angioplasty (PTA) and stent grafting were administered by the decision of the operator. For patients with concurrent embolism of the infrapopliteal artery, especially in the crural arteries, manual aspiration thrombectomy by appropriate end-hole catheters from 4–6 French was performed as the basic technique. Aspiration was performed by syringes with a volume of 20–50 ml. All procedures were performed by experienced endovascular specialists from our center.

## Results

We identified 17 patients who underwent PMT procedures with Rotarex S for ALE with a median follow-up of 22 months (range, 7–33 months). The mean age was 73 years (range, 54–90 years). Most patients were women (71%) with a high prevalence of typical vascular comorbidities, such as coronary artery disease (65%), hypertension (59%), hyperlipidemia (29%), history of stroke (29%), and current smoker (18%). The study population, anatomic segments treated, and outcomes are detailed in Supplementary Table S1. A total of 17 limbs were treated. Although one patient suffered from a bilateral embolism, he received PMT for the right limb, and the other side was managed conservatively. The most commonly treated vessels were the femoral and popliteal arteries, with both present in 59% of the patients, and only two patients had iliac arteries involved. Six patients had an infrapopliteal artery embolism, four of whom had embolus in the proximal infrapopliteal arteries treated by the Rotarex S device, and the remaining two patients had embolus in the distal infrapopliteal arteries, so manual aspiration was used.

The vast majority of patients were Rutherford II (88.2%), of which eight were Rutherford IIa, seven were Rutherford IIb, and the remaining two were Rutherford I. The time between the onset of ischemia and treatment varies from case to case. More than half of the patients received treatment within the first 24 h after the presentation.

Technical success was achieved in all patients with mechanical thrombectomy and additive simple aspirational thrombectomy from distal arteries. The majority of cases (82%) had concurrent PTA or stent grafting, and two patients were treated with urokinase during the operation because of the outflow obstruction for distal residual thrombi in tibial or pedal arteries. The average operation time and contrast amount used were 151 ± 44 min and 101 ± 26 ml, respectively, and the median blood loss was 100 ml (IQR, 50–200).

Intraoperative complications occurred in three patients (18%): two extravasations after PMT and one intraoperative distal embolization. Extravasations are located in the femoral artery and tibioperoneal trunk, respectively, and resolved with covered-stent graft implantation and long balloon inflation; no hematoma was detected after the operations. The distal embolization was cleared with manual aspiration by a 4-French catheter. Postoperative complications occurred in three patients (18%): one access site hematoma, one target artery thrombosis, and one acute kidney injury (AKI). The patient who underwent amputation was treated with PMT for popliteal artery and tibioperoneal trunk embolism, but it recurred 2 days later with popliteal artery thrombosis; this complication resulted in severe unsalvageable limb ischemia and required a major, below the knee, amputation. Neither the access site hematoma nor the AKI was serious, and all resolved with conservative treatment.

There were no 30-day deaths. All patients were subsequently treated with antithrombotic therapy and additional anticoagulation (rivaroxaban or warfarin). All patients attained clinical follow-up. One patient had recurrence and amputation as previously described; three patients died of unrelated causes during the follow-up.

## Discussion

Acute lower limb embolism is caused by reduced arterial perfusion to a limb due to embolic migration. In the general population, the incidence is estimated to be 14 per 100,000, with a highly morbid condition leading to 1-year mortality and amputation rates ranging between 16%–42% and 11%–37%, respectively (1, 10, 14). Similar to these cases, the heart has historically predominated as the most common source of all emboli, mainly from blood clots caused by paroxysmal atrial fibrillation and sometimes originating in the intracardiac mass (15, 16).

Urgent treatment to achieve revascularization of the ischemic limb is necessary, which may avoid the need for amputation and reduce mortality (3).

To achieve restoration of blood flow, the options for ALE patients include open revascularization and endovascular revascularization (17). For ALE patients, embolectomy through a transfemoral approach with a Fogarty balloon is the most common solution, and the direct popliteal approach or bypass surgery is also a surgeon’s choice (15, 18). However, although open surgery can restore blood flow exactly, making it recommended in ALE patients, especially those with advanced ischemia, there are still some disadvantages. First, frail ALE patients are usually associated with advanced cardiac disease and older age, and surgical trauma and general anesthesia bring higher complications, including wound infections, major cardiac events, and respiratory failure (17). Therefore, the mortality rates in a previous study reached 7%–10% (15, 19). Second, although the completion angiogram is also recommended in the ESVS guidelines for open surgery, surgical intervention is sometimes considered technically successful when a palpable pulse is detected at the completion of the procedure, and the patency of the distal vessels is unknown, which may lead to the recurrence of limb ischemia (9, 17).

With the improvement of treatment technology, new breakthroughs and advancements have been brought to the treatment of ALE, and endovascular treatment has reduced the physiological stress on frail ALI patients; somehow, there has been a dramatic increase in use and experience over the past decade (14, 17). In recent years, CDT has been one of the endovascular treatments with high success rates. However, CDT takes more time to restore the blood supply, and this disadvantage cannot be tolerated for patients with severe ischemia (IIb) requiring immediate revasularization. Meanwhile, although this locoregional infusion of thrombolytic medication can reduce major bleeding complications, the risk of hemorrhagic and even mortality rates is higher than PMT, and it is unfavorable in patients with contraindications of thrombolysis (3, 20, 21). On the other hand, the Rotarex S device can remove thrombi and emboli from the peripheral arterial system without thrombolytics, thereby reducing the risk of bleeding complications (3).

The Straub Rotarex S thrombectomy device works on the Archimedean screw principle; the catheter tip rotates at 40,000–60,000 rpm and fragments both the thrombi and emboli (6, 22). The fragments are then sucked into the catheter from the opening at the tip and transported out of the body with blood into a collecting bag (23). In this way, this rotational thrombectomy device may be an effective and safe modality for restoring blood supply to the target limb quickly and reducing thrombolysis. This highly efficient device showed encouraging technical success in our cohort, and the same results were seen in previous studies. The clinical success of using the Rotarex or Rotarex S device for ALI patients reached 94%–97% in different studies (24–26). These results were similar to those of earlier thrombolysis therapy, with clinical success rates ranging from 46% to 68% (27, 28). However, most of the studies on the Rotarex S device for the treatment of ALI or ALE are single-arm and retrospective, and they are inherently subject to selection bias, as we chose patients with a decreased degree of ischemia to undergo PMT. More research about direct comparison with open surgery and other endovascular treatment options should be conducted to demonstrate this advantage.

Another benefit of this treatment is its safety. A single cohort study for the Rotarex device vs. r-tPA in acute and subacute infra-aortic arterial occlusions patients found a higher 30-day limb salvage and fewer major vascular events (MVEs) in the Rotarex group (26). Another meta-analysis showed a similar result, with Rotarex having a significantly lower chance of enduring MVE (3.4%) than r-tPA (14%; *P* < 0.01) and a shorter duration of hospital stay in critically ill patients, but there was no significant difference regarding limb salvage in this study (1). Other PMT devices, such as Indigo (Penumbra Inc., Alameda, CA, United States), showed the same advantages as CDT (29). The AngioJet thrombectomy catheter also showed a lower overall mortality rate (21). In our study, only one patient had a small access site hematoma resolved conservatively. Hence, PMT may be ideal for patients with contraindications to thrombolytics or those with severe comorbidities who are not candidates for surgery. The use of PMT for ALE may carry other potential advantages. In our center, PMT is performed under local anesthesia or sedation, and complications caused by general anesthesia are rare. No major cardiac events, respiratory failure, or wound issues due to open surgery were observed in our patients.

However, it is worth noting that the Rotarex S device may have several potential drawbacks. First, this rotational thrombectomy device may include an increased risk of vessel injury. In particular, arterial perforation was frequently described in up to 10% of cases in early papers (26). In our cases, two patients had extravasation after using Rotarex; although these complications were solved with long balloon inflation and stent grafting without more serious consequences, it still showed the risk we mentioned above. The same concerns were shown in other studies. Heller et al. (23) showed that 5.5% of patients had extravasation or vessel perforation when using the Rotarex S device to treat ALI, and one patient even required surgical therapy. Vorwerk et al. (30) showed one arterial perforation in 60 cases. The risk of perforation would be higher in smaller and calcified arteries; typically, when a larger device is used, as in our study, both injured patients were using an 8F device (1, 6, 23). Second, owing to the design of the Rotarex device, there might be insufficient thrombi or emboli removal in larger vessels, such as the iliac artery. When some thrombi could be removed from the vessel, many stents were placed to avoid distal embolization (1). In our study, the only patients who needed stent grafting had iliac artery embolisms (except for the bailout stent). Finally, distal embolization is one of the particular issues with this device, which leads to the high cost or even severe unsalvageable limb ischemia. The possibility of thrombus and emboli fragmentation may result in intraprocedural distal embolization (3). The reported rate of distal embolization is approximately 4%–5%, and we encountered one patient in our cohort (5.9%) (23, 31). To avoid distal embolization, it is recommended to pass the occlusion slowly, and small forward and backward movements are performed (6). If distal embolization occurs, aspiration by manual or other devices, PTA, and local thrombolysis can be the choice depending on the situation and the operator's experience (3, 6).

A previous study suggested that open surgery may be the best option for ALI due to arterial embolism and for Rutherford IIB acute arterial thrombosis because of a greater urgency to restore blood flow (21). However, our study showed that PMT with the Rotarex S device appears to be a safe alternative for treating ALE with a low risk of complications and no 30-day deaths. However, additional treatment, such as PTA, is usually required to achieve a satisfactory restoration of blood flow.

Limitations: Our study has several notable limitations. This study is inherently subject to selection bias and a relatively small number of patients enrolled, as we chose patients with a decreased degree of ischemia to undergo endovascular treatment, and comorbid patients with irreversible limb ischemia classified as Rutherford III have proceeded to major amputation. We included only cases of the Rotarex S device, and the use of CDT or open surgery was not reviewed. In the future, a comparison with open surgery and other endovascular treatments is required to find the optimal treatment strategy. Second, although all patients received clinical follow-up, the duration of follow-up varied widely. However, this study was designed to evaluate the safety and limb salvage efficacy of this acute embolism management tool in treating ALE, not necessarily to prevent future long-term events.

## Conclusions

The Rotarex S device has a satisfactory success rate, although complementary use of various adjunctive techniques is frequently required. It seems to be a moderately effective tool for treating ALE to avoid CDT or open surgery. The device appears safe, with low risks of amputation and mortality rates, but special attention should be given to the potential for extravasation and distal embolism.

## Data Availability

The raw data supporting the conclusions of this article will be made available by the authors, without undue reservation.
