# Supplementary material for: Percutaneous mechanical thrombectomy using the Rotarex®S device for the treatment of acute lower limb artery embolism: A retrospective single-center, single-arm study
Source: Front Surg. 2023 Jan 6;9:1017045. doi: 10.3389/fsurg.2022.1017045 (PMC9859659; doi:10.3389/fsurg.2022.1017045)
Supplement: Supplementary file 1 [file Table1.docx]

Table 1 Study population and anatomic segments treated

| Patient number | Sex/age | severity of ALI | Onset(hour) | Causes of embolism | Vessel involvement | 6F/8F device used | Concurrent treatment | Complications (treatment) | Follow-up (month) | Outcome |
| --- | --- | --- | --- | --- | --- | --- | --- | --- | --- | --- |
| (1) | F/82 | IIb | 24 | AF | IA+SFA | 6F | PTA+stentgrafting | None | 4 | Completely recovered |
| (2) | M/60 | IIb | 13 | Atrial myxoma | SFA+PA+IPA (distal) | 6F | PTA+MA | None | 49 | Completely recovered |
| (3) | F/78 | IIa | 60 | AF | IA | 8F | PTA | None | 4 | Completely recovered |
| (4) | F/79 | IIa | 24 | AF | SFA+PA | 8F | PTA | None | 35 | Died |
| (5) | M/67 | IIa | 96 | AF | SFA+PA | 6F | Intraoperative urokinase | None | 23 | Completely recovered |
| (6) | F/59 | IIb | 20 | AF | PA+IPA (proximal) | 6F | PTA | None | 6 | Completely recovered |
| (7) | F/83 | IIa | 24 | AF | SFA+PA+IPA (proximal) | 8F | PTA | Extravasation (long balloon inflation ) | 22 | Completely recovered |
| (8) | M/87 | IIa | 10 | AF | PA+IPA (distal) | 6F | MA | None | 5 | Completely recovered |
| (9) | F/73 | IIa | 120 | Rheumatic heart disease | SFA | 8F | PTA+stentgrafting | Extravasation (stentgrafting) | 25 | Completely recovered |
| (10) | F/59 | IIb | 12 | AF | SFA | 8F | None | None | 31 | Completely recovered |
| (11) | F/83 | IIa | 120 | Undetermined | SFA | 8F | PTA+ MA | Distal embolization (MA) | 16 | Completely recovered |
| (12) | F/61 | I | 168 | AF | PA+IPA (proximal) | 6F | None | None | 40 | Completely recovered |
| (13) | F/81 | IIa | 168 | AF | PA | 6F | PTA | None | 37 | Completely recovered |
| (14) | F/74 | I | 240 | AF | SFA | 8F | PTA | Access site hematoma | 8 | Completely recovered |
| (15) | M/66 | IIb | 96 | Undetermined | PA+IPA (proximal) | 6F | PTA | Popliteal artery thrombosis (BKA) | 19 | Died |
| (16) | F/90 | IIb | 11 | Undetermined | SFA | 6F | PTA | None | 22 | Completely recovered |
| (17) | M/54 | IIb | 24 | Undetermined | PA+IPA (proximal) | 6F | PTA+Intraoperative urokinase | AKI (Hydration) | 10 | Died |

M: male; F: female; ALI: acute limb ischemia; AF: atrial fibrillation; IA: iliac artery; SFA: superficial femoral artery ; PA: popliteal artery; IPA: infrapopliteal artery; PTA: percutaneous transluminal angioplasty; MA: manual aspiration; BKA: below-knee amputation; AKI, acute kidney injury;

severity of ALI was determined based on Rutherford classification
